# Balancing access to ADHD medication

**DOI:** 10.1186/s12916-023-02917-4

**Published:** 2023-06-19

**Authors:** B.M.C. Medicine

**Affiliations:** The Campus, 4 Crinan Street, London, UK

Imagine going to your local pharmacy to pick up your repeat prescription and being told they are out of stock. You then hear the same news from every chemist in the area. That is the situation facing people with attention deficit hyperactivity disorder (ADHD), characterized by inattention, excessive impulsivity, and hyperactivity. Unlike other drug shortages caused primarily by supply chain problems, the reasons for this ongoing scarcity are more complicated.

Total psychostimulant prescriptions jumped 10% among American adolescents and adults in 2020–2021, the first year of the COVID-19 pandemic. This rise in ADHD diagnoses was attributed to the January 2020 suspension of the 2008 Ryan Haight Act by the USA Department of Health and Human Services, which strictly regulated the prescription of controlled substances through telepsychiatry. This emergency policy allowed telehealth providers to prescribe controlled substances online without needing in-person examination. This Act’s suspension meant there had never been an easier time to obtain psychostimulant prescriptions in the USA. Partially better awareness and detection of ADHD may also have played a role in increased diagnoses, driven by advances in diagnostic criteria, public awareness, and less stigmatization.

In October 2022, the Food and Drug Administration (FDA) announced a shortage of several Adderall formulations in the USA. Citing “intermittent manufacturing issues,” supply still cannot meet market demand 8 months on. Adderall, a mixed amphetamine salt, improves executive function and ameliorates ADHD symptoms. It is a medication essential for many to live normally and productively. Adderall withdrawal can severely affect one’s ability to function. Short supply has left many children and parents in turmoil, with reports citing significant school/work performance loss and turning to street drugs as illegal substitutes. Further deepening this problem, research indicates that the COVID-19 pandemic exacerbated the core symptoms of ADHD and comorbidities.

Compounding the shortfall of available medicines, exploitative companies are targeting those who do not truly need ADHD medication. One recent BBC investigation exposed the issue of private clinics lacking regulation, which led to inappropriate psychostimulant prescriptions in the UK. An undercover reporter revealed that three online clinics diagnosed him with ADHD using unreliable mental health assessments. The reporter was subsequently prescribed Ritalin for long-term use, a psychostimulant used to treat the condition. But a subsequent in-person NHS assessment showed that the journalist did not fit the diagnostic criteria for ADHD. Another investigation by ABC News found that psychostimulant prescriptions for ADHD in Australia had more than doubled in 2018–2023, with private clinics capitalizing and charging $3000 for online consultations. Both reports illustrate potential ADHD misdiagnosis and prescription misuse in these countries.

American telemedicine start-ups have been accused of prioritizing profit by former employees and government lawyers. Some are under federal investigation for violating the Controlled Substances Act. Most early pandemic prescribers have either ceased operations or had their licenses revoked, yet the number of Americans officially diagnosed with ADHD remains in the millions. Psychostimulant prescriptions are for life, meaning that they have no expiry date. In a letter to drugmakers last summer, the FDA pointed to “the sheer volume of ADHD medications on the market coupled with aggressive marketing practices” has led to a shortfall in the supply of ADHD medications.

Government regulators have known about a potential shortfall of Adderall in the USA since 2021 but have been slow to act. The FDA announced plans to update box labels to curb misuse by guiding clinicians and patients, but this does nothing to address Adderall’s short supply. Psychostimulants used to treat ADHD are designated as schedule II controlled substances, so they possess therapeutic use but come with “a high potential for abuse.” The Drug Enforcement Administration (DEA) regulates production so that there is just enough supply while minimizing the risk of misuse or addiction. Monitoring rules on “suspicious orders” are proposed to become stricter under a proposed draft. Psychostimulant orders with unusual features would be flagged to the DEA so customers could be reported to this law enforcement agency for investigation. Though this move would prevent potentially inappropriate prescriptions, misuse of this proposed policy would add yet another barrier to legitimate patient access.

The number of child and adolescent psychiatrists is low, and their patients are one of the most underserved populations—fewer than ten specialists per every 100,000 children in the USA. New prescriptions must be obtained if one’s existing pharmacy runs out of stock, disproportionately discriminating against those who do not own private health insurance. In the US Congress, Rep. Abigail Spanberger pressed the FDA to solve this public health emergency. However, the FDA’s outlook looks like one of law enforcement fearful of a repeat of another opioid epidemic.

Pandemic changes shifted the pendulum on ADHD medication supply to trigger shortages in an already tightly restricted market. Lack of access to Adderall for patients is an unnecessary crisis that needs swift action. There is a need to tackle misuse by regulating private providers and increasing funding for child and adolescent psychiatrists in the medium to long term. Additionally, ensure that short-term production and distribution constraints are adjusted to guarantee appropriate providers are fully supplied with medication. A balance between prohibitive policies protecting patients and providing reasonable access to ADHD medication must be a public health priority.

This editorial highlights the vital issue of fair access to diagnosis/treatment during May’s mental health awareness month. *BMC Medicine* recently announced a call for papers welcoming submissions on neurodevelopmental disorders. This article collection plans to focus on often underserved, misunderstood conditions and patients.

## Abbreviations

ADHD: Attention deficit hyperactivity disorder
DEA: Drug Enforcement Agency
FDA: Food and Drug Administration

